# Mitogenome of a stink worm (Annelida: Travisiidae) includes degenerate group II intron that is also found in five congeneric species

**DOI:** 10.1038/s41598-022-08103-5

**Published:** 2022-03-15

**Authors:** Genki Kobayashi, Hajime Itoh, Shigeaki Kojima

**Affiliations:** 1 Seto Marine Biological Laboratory, Field Science Education and Research Center, Kyoto University, 459 Shirahama, Nishimuro, Wakayama, 649-2211 Japan; 2 National Institute for Environmental Studies, 16-2 Onogawa, Tsukuba, Ibaraki 305-8506 Japan; 3 Atmosphere and Ocean Research Institute, The University of Tokyo, 5-1-5 Kashiwanoha, Kashiwa, Chiba 277-8564 Japan

**Keywords:** Phylogenetics, Evolutionary biology, Genomics

## Abstract

Mitogenomes are useful for inferring phylogenetic relationships between organisms. Although the mitogenomes of Annelida, one of the most morphologically and ecologically diverse metazoan groups have been well sequenced, those of several families remain unexamined. This study determined the first mitogenome from the family Travisiidae (*Travisia sanrikuensis*), analyzed its mitogenomic features, and reconstructed a phylogeny of Sedentaria. The monophyly of the Terebellida + Arenicolida + Travisiidae clade is supported by molecular phylogenetic analysis. The placement of Travisiidae is unclear because of the lack of mitogenomes from closely related lineages. An unexpected intron appeared within the *cox1* gene of *T. sanrikuensis* and in the same positions of five undescribed *Travisia* spp. Although the introns are shorter (790–1386 bp) than other group II introns, they can be considered degenerate group II introns due to type II intron maturase open reading frames, found in two of the examined species, and motifs characteristic of group II introns. This is likely the first known case in metazoans where mitochondrial group II introns obtained by a common ancestor are conserved in several descendants. Insufficient evolutionary time for intron loss in Travisiidae, or undetermined mechanisms may have helped maintain the degenerate introns.

## Introduction

The mitochondrial genome (mitogenome) has become commonly used for molecular phylogenetic analysis. Although mitogenomic phylogeny is less informative for resolving the higher classification, it often yields a robust framework for the phylogenetic relationships on shallow nodes^1,2^. In addition to phylogenetic reconstruction based on the nucleotide sequences of mitogenomes, gene order rearrangement has been used for inferring phylogenetic relationships^3,4^. The gene order of a mitogenome is relatively conserved; the order is sometimes the same among higher taxa, e.g., across orders of annelids, when considering only protein-coding genes (PCGs)^3^. Conversely, the gene order in some marine invertebrates, including annelids, shows an intra-familial variation^2,5–9^ and may shed light on the phylogenetic relationships of relatively closely related taxa.

Mitochondrial DNA (mtDNA) is a closed-circular molecule in most animals and is generally small (15–20 kb) compared to the nuclear genome. Animal mtDNA usually contains 37 genes, namely 13 PCGs (*cox1–3*, *atp6*, and 8, *cytb*, and *nad1–6* and *nad4l*), 22 tRNAs, and two rRNAs^10^. Non-coding regions within the PCGs (i.e., introns) of mtDNA are known for many eukaryotes^11^. Known mitochondrial introns are mainly classified as groups I and II based on their structural features^11^. The group I introns are predominant in fungi, whereas group II introns are most frequent in plants^12^. Both group I and II introns appear to be rare in metazoan mitogenomes^13^. Indeed, reports of metazoan species possessing mitochondrial group II introns are sporadic. At least seven species in four phyla, namely Porifera^13^, Placozoa^14^, Mollusca^15^ (see “Discussion”), and Annelida^16–18^, possess group II introns in their mitogenomes. Group II introns are generally characterized by a secondary structure with six domains and intronic open reading frames (ORFs), encoding functions for splicing and mobility (e.g., reverse transcriptase and RNA maturase), and motifs beginning with 5′ GUGYG 3′ and ending with 5′ AY 3′^11,19,20^. However, these features are not necessarily present in all group II introns; for example, ORF-less introns^21^ and nucleotide substitutions in characteristic motifs^22^ have also been reported.

The phylum Annelida has over 20,000 described species^23^, including polychaetes, echiurans, sipunculans, leeches, and oligochaetes. The annelids show high morphological and ecological trait diversity and have adapted to various environments ranging from terrestrial sites to the ocean’s hadal zones. They are therefore interesting subjects for evolutionary study. The phylogenetic relationships between a wide range of lineages in Annelida have been well assessed using expressed sequence tags^24^, transcriptomic data^25–29^, and mitogenomes^3,30–32^. Currently, two major groups (Errantia and Sedentaria) and some early-branching families are recognized in Annelida. Sedentaria includes echiurans, vestimentiferans, clitellates (leeches and oligochaetes), and the sessile and tube-dwelling polychaetes. Several polychaete families are not yet included in the phylogenomics of annelids and therefore inter-familial relationships remain to be fully understood^33^.

**Figure 1:**
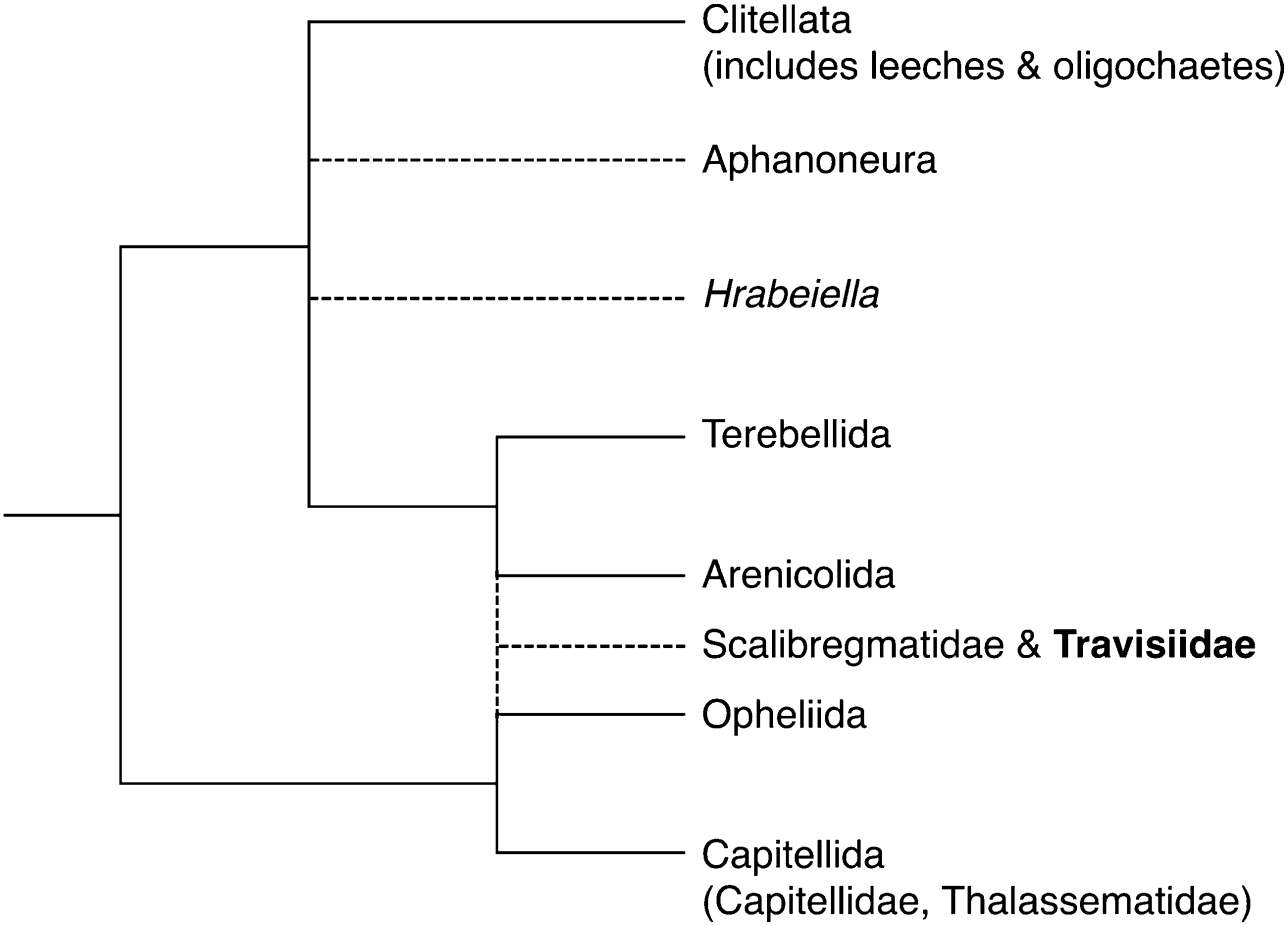
Phylogenetic relationships from a subset of Sedentaria modified from a metatree regarded as a working hypothesis for future studies by Struck^33^. Dashed lines indicate lineages with undetermined phylogenetic positions.

The family Travisiidae includes small vermiform annelids with a single valid genus, *Travisia*, and at least 37 described species^34–37^. The species of *Travisia* are deposit feeders inhabiting the muddy bottom mainly in deep-sea lower than 200 m depth (reviewed by Blake and Maciolek^35^). The presence of *Travisia* in sediment samples is noticeable by their characteristic fetid odor and *Travisia* are known as “stink worm” for the smell. Although the function of chemical substances that are the source of the odor, is not fully understood, Taboada et al.^38^ verified that the lipophilic extract of *Travisia* sp. deters predatory starfish (the authors say it needs careful interpretation), and Nara and Seike^39^ inferred from the aggregation of trace fossil *Macaronichnus segregatis degiberti* that volatile chemical substances of *Travisia* might act as sex pheromones. Penry and Jumars^40^ hypothesized that microbial fermentation may be important in the digestive strategy of *T*. *foetida* considering the odor and the unusual gut structure of this species. Previously, *Travisia* and two synonymized genera (*Dindymenides* and *Kesun*^41^) were considered, based on morphological characters (see Rouse^42^), to be members of Opheliidae, which clusters with Capitellida by molecular phylogenetic analysis^26^ (Fig. 1). Conversely, molecular phylogeny indicates a close relationship between *Travisia* and scalibregmatid species, not Opheliidae^43^, and Travisiidae was recognized as a distinct subgroup in Scalibregmatidae^44–47^. The subgroup has been considered independent and raised to the family level based on morphological evidence^35^. Scalibregmatidae is clustered with Terebellida + Arenicolidae clade in recent phylogenomics based on transcriptomes^48^.

In this study, we determined the mitochondrial genome sequence of *Travisia sanrikuensis*, the first mitogenome from the family Travisiidae, to elucidate the species’ mitogenomic features, reconstruct the phylogeny of Sedentaria, and examine the phylogenetic position of Travisiidae. The features of the mitochondrial genome, the intron in the barcoding region of *cox1*, and gene rearrangements are discussed. In addition, the nucleotide sequences of the mitochondrial *cox1* intron of *Travisia* spp. were determined, and phylogenetic analysis was performed using the partial sequences of the group II intron.

## Results

### Assembly of the mitogenome

A total of 474,608 reads were obtained after trimming low-quality reads. A merged contig for *T. sanrikuensis* (12,166 bp) was obtained from an initial NOVOPlasty run using the 16S rRNA gene sequence (LC566242) seed. Although several assembly conditions were tested by varying kmer and read length, a merged contig longer than 500 bp was obtained only with kmer and read length set to 23 bp and 111 bp, respectively. A region from the merged contig showed moderately high homology (785 bp, max score 250, total score 665) to the *nad5* gene of *Glycera* cf. *tridactyla* (KT989327) during a BLAST homology search. A partial sequence (192 bp) from the predicted *nad5* gene in the initial *T. sanrikuensis* contig, which aligned with the *nad5* gene of *G.* cf. *tridactyla* (KT989327) from position 6219–6410, was used as a seed sequence for a subsequent assembly. The resulting merged contig was 17,390 bp in length. Both ends of the contig had a consensus sequence larger than 100 bp, with both ends of the 16S rRNA gene sequence used as the initial seed (LC566242). Although the circular mitogenome of *T. sanrikuensis* was recovered by concatenating the contig and 16S rRNA gene sequence (LC566242), a dubious control region (> 2000 bp) between the *nad5* and *trnR* genes, which includes tRNAs encoded on “−” strand and a long palindrome like sequence (a nearly perfect inverted repeat of > 600 bp), was present. This control region should be confirmed by polymerase chain reaction (PCR) but PCR failed to amplify a target including the control region and therefore the nearly complete mitogenome sequence (15,854 bp), excluding the control region, was registered (LC677172).

### Mitochondrial genome organization

**Figure 2:**
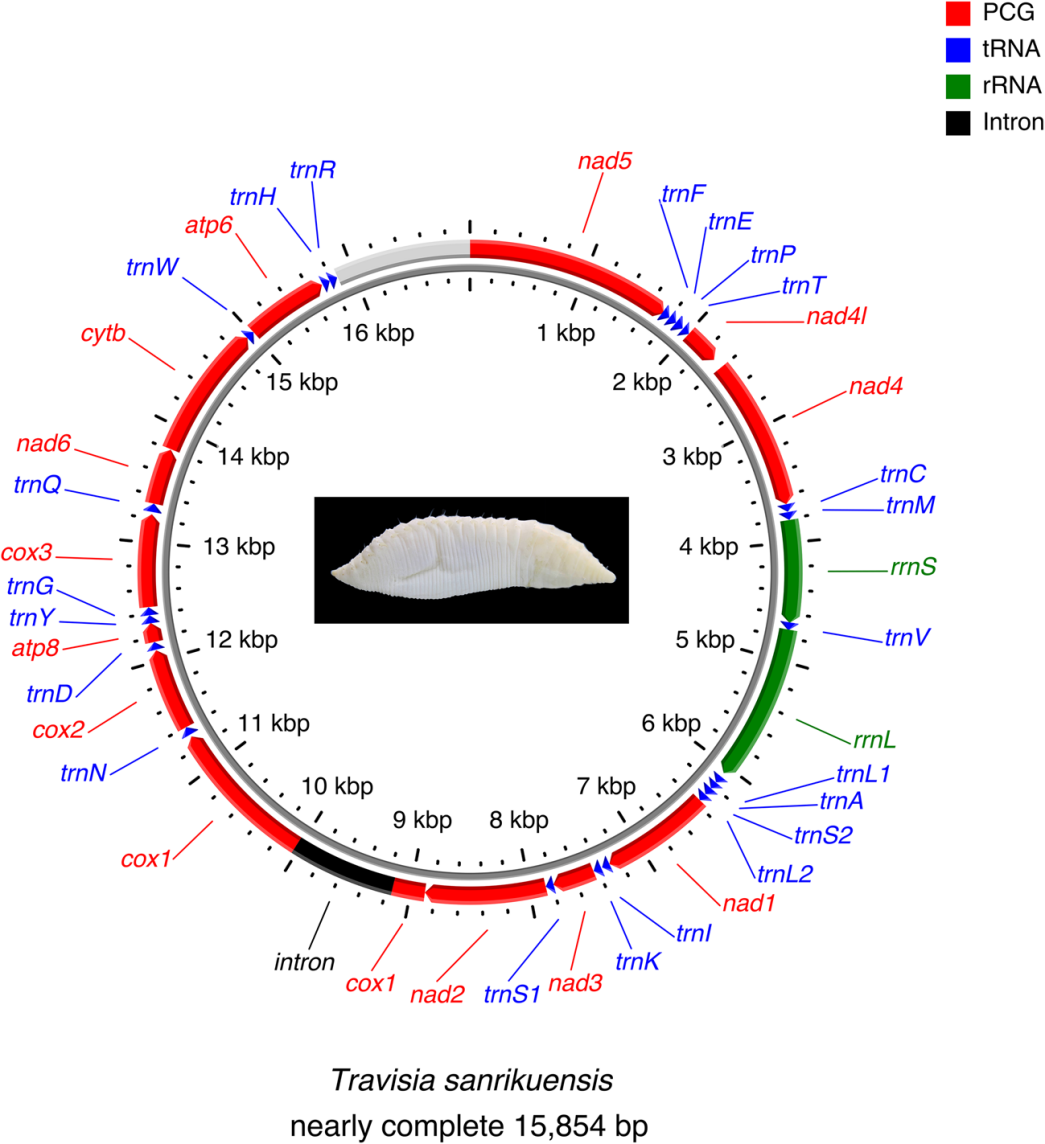
Gene map of the nearly complete mitochondrial genome of *Travisia sanrikuensis*. A photograph shows *T*. *sanrikuensis*. Red: protein-coding genes, Blue: tRNAs, Green: rRNAs, Black: intron, Light gray: undetermined positions including a putative control region.

The mitogenome sequence includes 13 PCGs (*atp6* and *8*, *cox1–3*, *cytb*, *nad1–6* and *nad4l*), 22 tRNA genes (one for each of the amino acids except for *trnL* and *trnS*), two rRNA genes [small ribosomal RNA (*rrnS* or 12S rRNA) and large ribosomal RNA (*rrnL* or 16S rRNA)] (Fig. 2 and Table 1). All determined genes were encoded on the “+” strand (Fig. 2). Both AT-skew and GC-skew of all genes, except for AT-skew of *rrnS*, were negative, indicating that T and C outnumber A and G, respectively (Table 2). Predicted secondary structures of tRNAs showed that the thymidine loops of *trnD*, *trnM,* and *trnI* and the dihydrouridine loop of *trnK* were reduced by 3 bp (Dataset S2). Dihydrouridine stem was lost in *trnS1* (Dataset S2).

**Table 1:** Summary of the nearly complete mitochondrial genome of *Travisia sanrikuensis* (15,854 bp).

Gene^a^	Position	Length (bp)	Strand	Codons^b^
*nad5*	1–1738	1738	+	ATT/T(AA)
*trnF* (GAA)	1726–1797	72	+	
*trnE* (TTC)	1799–1867	69	+	
*trnP* (TGG)	1866–1940	75	+	
*trnT* (TGT)	1945–2009	65	+	
*nad4l*	2010–2312	303	+	ATG/TAA
*nad4*	2399–3677	1279	+	ATA/T(AA)
*trn*C (GCA)	3678–3742	65	+	
*trnM* (CAT)	3745–3811	67	+	
*rrnS*	3812–4676	865	+	
*trnV* (TAC)	4672–4739	68	+	
*rrnL*	4733–6062	1330	+	
*trnL1* (TAG)	6100–6163	64	+	
*trnA* (TGC)	6165–6228	64	+	
*trnS2* (TGA)	6229–6296	68	+	
*trnL2* (TAA)	6299–6365	67	+	
*nad1*	6365–7295	931	+	ATG/T(AA)
*trnI* (GAT)	7296–7362	67	+	
*trnK* (TTT)	7372–7439	68	+	
*nad3*	7442–7795	354	+	ATG/TAA
*trnS1* (TCT)	7794–7861	68	+	
*nad2*	7862–8879	1018	+	ATT/T(AA)
*cox1*	8880–9151, 10,034–11,309	1548	+	ATG/TAA
Group II intron	9152–10,033			
*trnN* (GTT)	11,329–11,398	70	+	
*cox2*	11,399–12,091	693	+	ATG/TAA
*trnD* (GTC)	12,097–12,162	66	+	
*atp8*	12,163–12,324	162	+	ATG/TAG
*trnY* (GTA)	12,322–12,389	68	+	
*trnG* (TCC)	12,390–12,457	68	+	
*cox3*	12,459–13,238	780	+	ATG/TAA
*trnQ* (TTG)	13,258–13,329	72	+	
*nad6*	13,329–13,802	474	+	ATT/TAA
*cytb*	13,807–14,946	1140	+	ATG/TAA
*trnW* (TCA)	14,945–15,014	70	+	
*atp6*	15,015–15,713	699	+	ATG/TAA
*trnH* (GTG)	15,721–15,786	66	+	
*trnR* (TCG)	15,787–15,854	68	+	

^a^Anticodons of tRNA are shown in parentheses.

^b^Incomplete stop codons are shown in parentheses.

**Table 2:** Nucleotide composition (%) of 13 protein-coding genes and rRNAs, and the skewness of *Travisia sanrikuensis*.

	Length	A	C	G	T	A + T	AT-skew	GC-skew
*atp6*	699	26.3	22.6	9.4	41.6	68.0	− 0.23	− 0.41
*atp8*	162	23.5	29.0	6.8	40.7	64.2	− 0.27	− 0.62
*cox1*	1548	26.7	22.2	15.5	35.5	62.3	− 0.14	− 0.18
*cox2*	693	29.0	24.4	13.9	32.8	61.8	− 0.06	− 0.28
*cox3*	780	25.3	22.7	15.8	36.3	61.5	− 0.18	− 0.18
*cytb*	1140	27.1	24.1	12.5	36.2	63.3	− 0.14	− 0.32
*nad1*	931	26.0	21.9	11.4	40.7	66.7	− 0.22	− 0.32
*nad2*	1018	27.2	25.2	8.8	38.7	65.9	− 0.17	− 0.48
*nad3*	354	24.6	23.7	11.0	40.7	65.3	− 0.25	− 0.37
*nad4*	1279	25.5	24.6	9.3	40.7	66.1	− 0.23	− 0.45
*nad4L*	303	24.8	20.8	12.9	41.6	66.3	− 0.25	− 0.24
*nad5*	1738	27.8	23.9	9.4	38.9	66.7	− 0.17	− 0.44
*nad6*	474	25.1	21.5	8.4	44.9	70.0	− 0.28	− 0.44
*rrnL*	1330	32.1	17.7	13.5	36.7	68.8	− 0.07	− 0.14
*rrnS*	865	32.4	22.7	15.5	29.5	61.8	0.05	− 0.19

**Figure 3:**
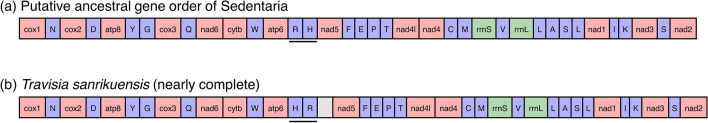
Gene order of the mitochondrial genome of (**a**) the putative ancestral gene order of Sedentaria (known for oligochaetes, leeches, and Siboglinidae) and (**b**) the nearly complete sequence of *Travisia sanrikuensis*. Red: protein-coding genes, Blue: tRNAs, Green: rRNAs, Gray: not determined. Underlines indicate gene order that differs between (**a**) and (**b**).

Figure 3 shows the gene order of *T. sanrikuensis* and the putative ancestral gene order of PCGs. The gene order was identical to the order commonly found among Errantia and Sedentaria. The gene order, including determined tRNAs, was almost identical to the putative ancestral gene order of Sedentaria, which is known for oligochaetes, leeches, and Siboglinidae^31,32^ but the order of *trnR* and *trnH* diferred between *T*. *sanrikuensis* and the ancestors of Sedentaria.

### Features of the *cox1* gene sequence in species of *Travisia*

The *cox1* gene of *T. sanrikuensis* included an intron (882 bp) within the “Folmer region” and thus possessed a longer target sequence (1540 bp) than usual (658 bp). PCR successfully amplified the partial *cox1* sequences of five unidentified species of *Travisia* and all of them included an intron (Table 3). The length of the introns of four *Travisia* spp. (*T. sanrikuensis*, GK623, GK625 and GK1734) were of varying lengths (790–1386 bp), although the intron sequences of two *Travisia* spp. (GK1732 and GK1736) were only partially determined (Dataset S3). The fully determined introns of *Travisia* spp. are shorter than the known mitochondrial *cox1* introns in annelids (1647–2468 bp)^16–18^. The introns were inserted at the same positions in all specimens of *Travisia*. Sequence logos identified several conservative regions (Fig. S2).

**Table 3:** Intron size of *Travisia* spp.

Species	Intron size (bp)
*Travisia sanrikuensis*	882
*Travisia* sp. GK623	865
*Travisia* sp. GK625	1386
*Travisia* sp. GK1732	> 861^a^
*Travisia* sp. GK1734	790
*Travisia* sp. GK1736	> 1156^a^

^a^Partial nucleotide sequences were determined.

The obtained nucleotide sequences in the “Folmer region” of *Travisia* were longer than expected, and thus the sequences were compared with those registered in the NCBI database. The BLAST search using *cox1* from *T. sanrikuensis* did not return any sequences of *Travisia* (HM473706–HM473709, HQ025027, HM904906, and MF121290). The BLAST search with *Travisia pupa* sequences (HM473706–HM473709) resulted in a low max score (≤ 95.3), whereas the results of a search using *T*. *sanrikuensis* returned the mitogenome sequence of annelid species *Melinna cristata* (Ampharetidae; MW542504; max score = 926). Only five sequences were returned by the BLAST search of *Travisia forbesii* (HQ025027, HM904906, and MF121290), while 100 metazoan sequences were returned for *T*. *sanrikuensis*. An alignment of two scalibregmatids sequences (JN256052 and MN217515) and sequences from *Travisia* species showed ambiguous indels in the sequences of *T. pupa* (HM473706–HM473709), including indels that do not correspond to triplets (Dataset S4).

### Introns in the *cox1* gene of *Travisia* spp

**Figure 4:**
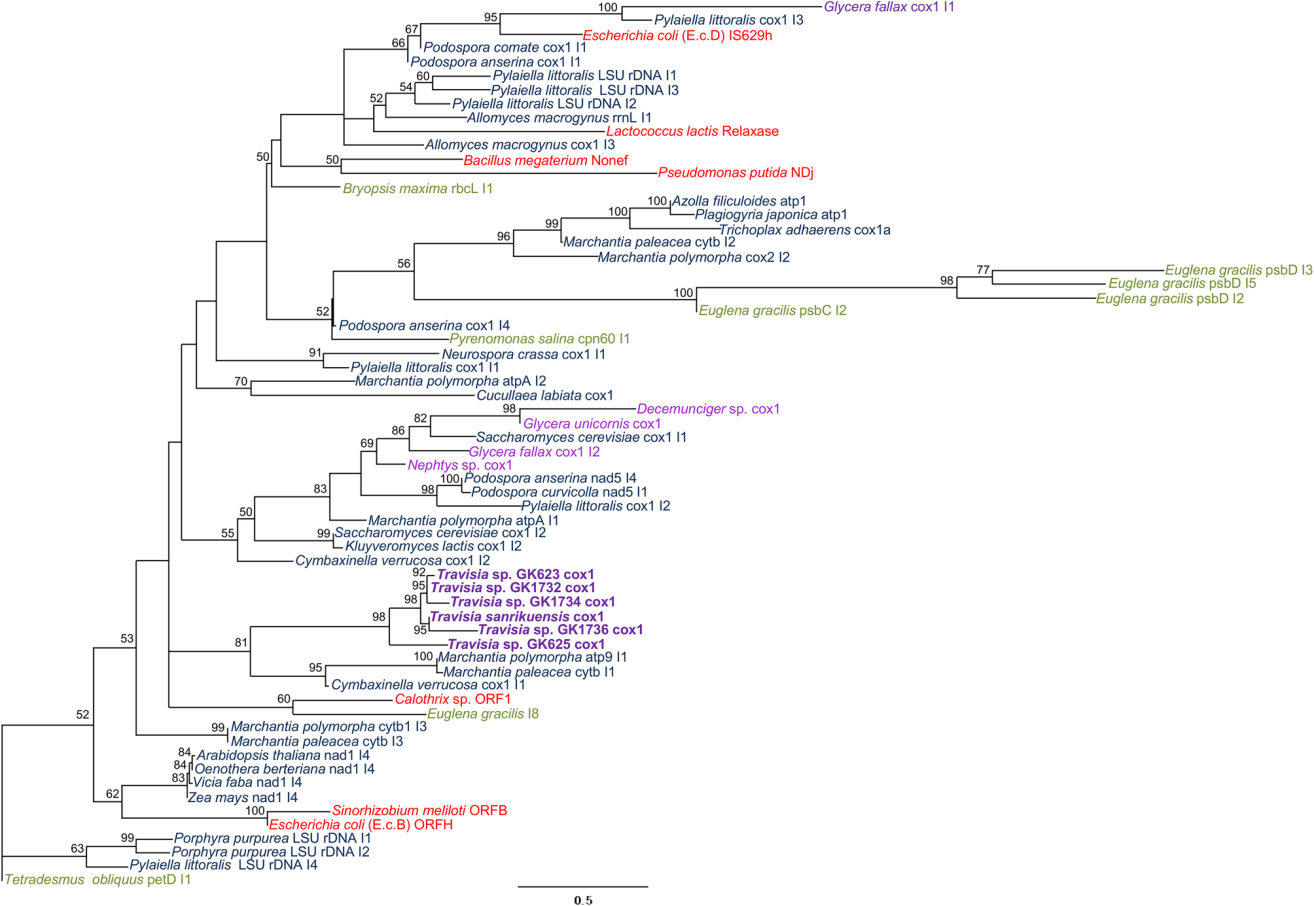
Maximum likelihood phylogeny of group II intron based on the nucleotide sequences of domain V and subsequent sites. The percentage of maximum likelihood bootstrap values (BS) ≥ 50% is shown above branches. Scientific names are followed by the host gene and intron ID. Bacterial group II introns (red), chloroplasts (green), and mitochondrial (blue) group II introns are included in the analysis. Annelid mitochondrial introns are shown in purple. OTUs with newly obtained sequences are in bold.

The introns of *Travisia* spp. begin and end with motifs that are characteristic of group II introns (5′ GCGCG 3′ and 5′ AY 3′, respectively). Mfold identified secondary structures corresponding to domains V and VI of group II introns but other domains were not recovered. ORFs for type II intron maturase, characteristic of group II introns, were found in two species, namely *Travisia* sp. GK625 and *Travisia* sp. GK1736, by PfamScan. Phylogenetic analysis based on domain V and subsequent sequences of group II intron showed that *Travisia* spp. introns were monophyletic (BS = 98%) (Fig. 4). This clade did not cluster with the group II introns of other annelids, i.e., *Decemunciger* sp., *Nephtys* sp., *Glycera fallax* (*cox1* I1 and I2), and *Glycera unicornis*. These annelid introns, except for *G*. *fallax cox1* I1, were closely related and *G*. *unicornis* and *Decemunciger* sp. introns were monophyletic (BS = 98%). The intron of *G*. *fallax cox1* I1 was not related to annelid introns but was clustered with the intron of the brown alga *Pylaiella littoralis* (BS = 100%). Sequence logos indicated five regions in the intron dataset were conservative and they roughly corresponded to the positions in the stem and ζ′ in the loop of domain V, and the stem of domain VI (Fig. S3).

### Phylogenetic relationships based on mitogenome sequences

**Figure 5:**
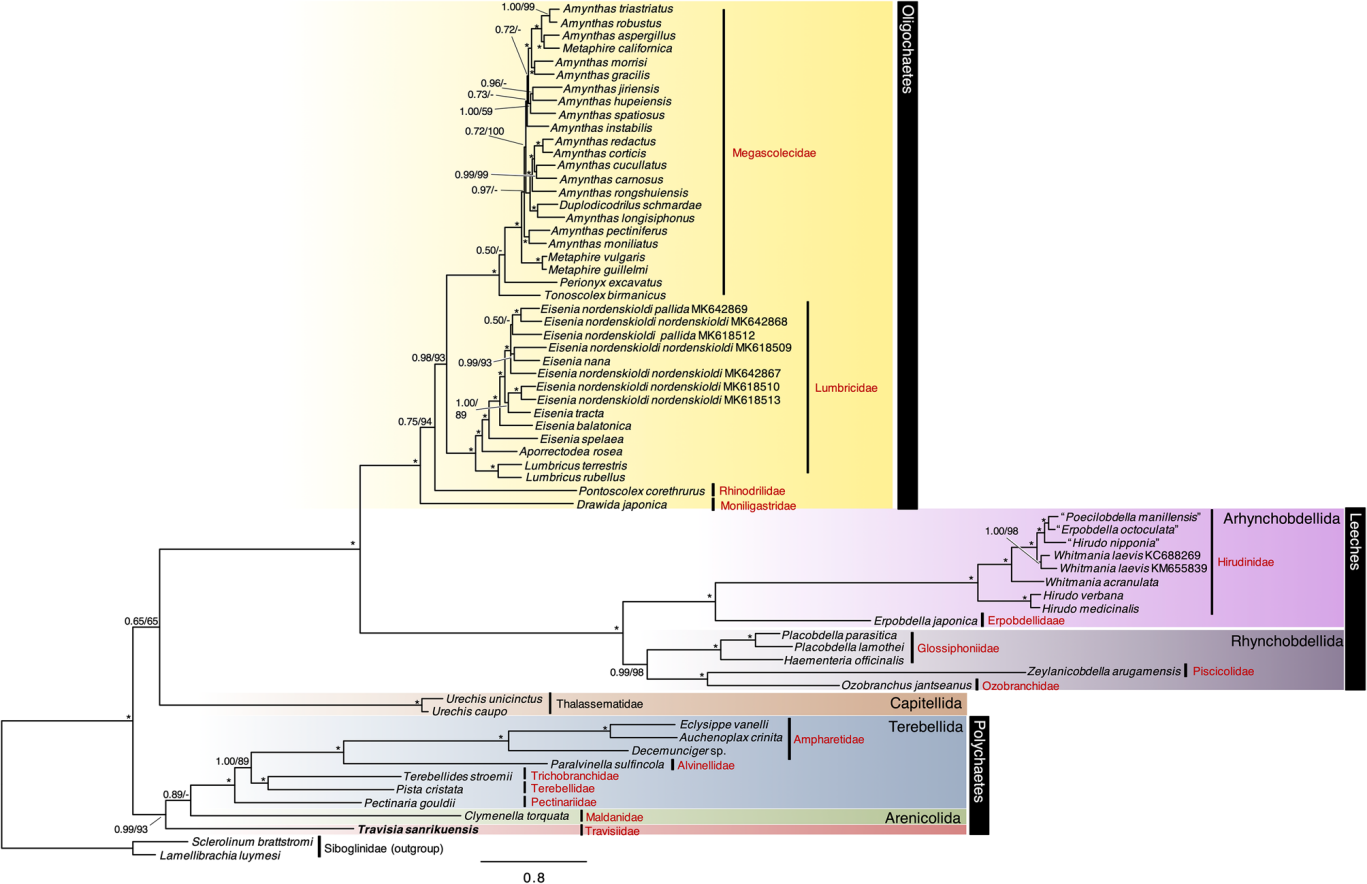
Bayesian phylogeny of a subset of Sedentaria based on the concatenated dataset, including the nucleotide sequences of 13 mitochondrial genome PCGs, 16S rRNA, and 12S rRNA (12,732 characters). Posterior probability (PP) followed by the percentage of the maximum likelihood bootstrap values (BS) above 50% is shown as numbers above branches. Asterisks indicate PP = 1.00 and BS = 100. *Travisia sanrikuensis*, for which the nucleotide sequence was newly obtained, is shown in bold.

*Travisia sanrikuensis* was included in the Maldanidae + Terebellida cluster with high support values (nucleotide: PP = 0.99, BS = 93%; AA: PP = 1.00, BS = 100%) but did not cluster with Thalassematidae in both nucleotide and AA sequence-based analyses (Fig. 5, Fig. S4). The monophyletic Terebellida clade was recovered as follows in the Newick format: (Pectinariidae, ((Terebellidae, Trichobranchidae), (Alvinellidae, Ampharetidae))). The phylogenetic positions of Thalassematidae (Capitellida) and *Travisia* were incongruent between nucleotide and AA sequence-based analyses. In the nucleotide-based analysis, Thalassematidae clustered with oligochaetes although support values were low (PP = 0.65, BS = 65%) (Fig. 5). *Travisia sanrikuensis* was sister to the clade Arenicolida (Maldanidae in the present analyses) + Terebellida (Ampharetidae, Alvinellidae, Pectinariidae, Terebellidae, and Trichobranchidae) but the support value of this lineage was low (PP = 0.89) and was not recovered by maximum likelihood (ML) analysis. In the AA-based analysis, the monophyly of early-branching Thalassematidae and polychaetes, including newly sequenced *T. sanrikuensis*, had relatively high support (PP = 0.98, BS = 94%) (Fig. S4).

All leech nodes were highly supported (PP ≥ 0.99, BS ≥ 98%) (Fig. 5). Rhynchobdellida (proboscis-bearing leeches) was recovered as monophyletic (PP ≥ 0.99, BS ≥ 98%). In Rhynchobdellida, Glossiphoniidae was sister to monophyletic Oceanobdelliformes (Ozobranchiidae and Piscicolidae). Monophyletic Arynchobdellida (leeches without a proboscis) (PP = ﻿1.00, BS = 100%), including Erpobdellidae and Hirudinidae, was sister to Rhynchobdellida. Support values in the oligochaetes were largely low and this group was not the main subject of the present study, and thus, phylogenetic relationships in oligochaetes have not been mentioned here.

## Discussion

We determined the nearly complete mitogenome sequence of a species from Travisiidae for the first time. Unexpectedly, an intron of a relatively short length (882 bp) was identified in the *cox1* gene of *T. sanrikuensis*. Introns were also found in five undescribed travisiid species using Sanger sequencing. All determined travisiid introns in the mitochondrial *cox1* gene (ranging from 790–1386 bp) were shorter than known *cox1* introns found in Annelida, i.e., 1819 bp in *Nephtys* sp., 2357–2468 bp in *Glycera* spp., and 1647 bp in *Decemunciger* sp. The introns of travisiid species included motifs (beginning with 5′ GCGCG 3′ and ending with 5′ AY 3′) and domains V and VI that are characteristics of group II introns. Also, the ORFs for type II intron maturase, found in two *Travisia* spp. (GK625 and GK1736), are the characteristics of mitochondrial group II introns found in annelids^16,17^. Travisiid introns were inserted in the same position across species. They formed a monophyletic group, suggesting that an intron with an ORF was obtained in a common ancestor of *Travisia* and the ORF was subsequently lost in some travisiid species. We regarded travisiid introns as degenerate group II introns based on these lines of evidence. ORF-less introns have been found in bacteria^21^ and fungus^49^. Also, although the *cox1* intron in the bivalve *Cucullaea labiata*^15^ is short (651 bp; positions 1184–1834 of KP091889) and lacks ORFs, it probably belongs to group II, considering the motifs at the 5′ (5′ GTGCG 3′) and 3′ ends (5′ AT 3′), and conserved regions suggested by the sequence logos (Fig. S3).

It is noteworthy that an intron was detected in all successfully sequenced travisiids in this study, considering that introns presumedly possess a high loss rate during speciation^16^. Richter et al.^17^ showed an absence of group II introns in *Glycera nicobarica*, which is closely related to *G*. *fallax* and *G*. *unicornis* (*G*. *fallax*, (*G*. *nicobarica*, *G. unicornis*)). The group II introns were probably obtained in a common ancestor of *Travisia* and have remained conserved (see above). Two possible scenarios explain the retention of the introns in *Travisia* spp.: (1) *Travisia* radiated rapidly, and thus had insufficient time to lose the intron from *cox1*. Indeed, the relatively small diversity of Travisiidae, with a single genus and about 40 described species, supports recent speciation of the group; (2) undetermined mechanisms help maintain the *cox1* intron travisiid species. Unfortunately, it is difficult to test these hypotheses at this stage. The robust phylogenetic framework of travisiid species and knowledge of the mitochondrial intron's function are needed to further discuss the evolutionary history of the degeneration of the travisiid mitochondrial intron. Nevertheless, *Travisia* is a promising subject for studying the loss and gain of mitochondrial introns.

The introns of *Travisia* spp. were inserted within the “Folmer region” of the *cox1* gene and this may have prevented amplification of *cox1* due to short amplification times during PCR. Only seven sequences of the *cox1* gene, which are obtained in DNA barcoding studies^50,51^, are available on GenBank: *T. forbesii* (HQ025027, HM904906, and MF121290) and *T. pupa* (HM473706–HM473709). However, the results of BLAST and alignment with scalibregmid sequences (MN217515 and JN256052) and *T*. *sanrikuensis* suggests that the *cox1* sequences registered as belonging to *Travisia* are not likely derived from *Travisia*. The possibility of contamination of the *cox1* sequences of *Travisia* in GenBank has been previously discussed (see the caption of Fig. 3 in Sun et al.^52^).

The phylogenetic relationships of leeches were contentious since the phylogenies based on several mitochondrial and nuclear genes were often incongruent^53–55^. Although phylogenomic studies with limited taxon sampling of annelids showed Rhynchobdellida as paraphyletic^56,57^, phylogenomic analysis based on anchored hybrid enrichment^58^ and transcriptomes^28^ with more taxon sampling revealed the monophyly of Rhynchobdellida. The high support for relationships among families in leeches in our results provides further support for the monophyly of Rhynchobdellida. On the other hand, the number of families in Arhynchobdellida represented by mitogenomes remains limited for proper phylogenomic studies. Therefore, further taxon sampling is needed to confirm the monophyly of hirudinean orders.

The relationships of polychaetes and clitellates ((Terebellida, Arenicolidae), clitellates) are consistent with previous phylogenomic studies^25,26^. The phylogenetic relationship within Terebellida is consistent with the recently published tree based on transcriptomes on Terebellida^59^ except for Melinnidae, whose mitogenome sequence is not included in this study. We confirmed the monophyly of *Travisia*, Terebellida, and Arenicolida (Fig. 5). The close relationship between *Travisia*, Arenicolida, and Terebellida was similar to the relationship (Scalibregmatidae, (Arenicolida, Terebellida)) in phylongeny based on 18S rRNA gene sequences^60^ and phylogenomics^48^, considering the sister relationship between *Travisia* and Scalibregmatidae^44–47^. Close relationships between Arenicolida and Scalibregmatidae + *Travisia*^61^ and Terebellida and Arenicolida^25,26,60,62^ has also been indicated previously. The morphological characters shared among the families in Arenicolida + Terebellida + *Travisia* (summarized in Rouse and Fauchald^63^, Appendix I and II) are also found in other lineages; therefore, no synapomorphy is known at this moment for this clade.

In the *Travisia* + Arenicolida + Terebellida clade, intra-familial molecular phylogenetic analyses have been conducted for Arenicolidae^64,65^, Maldanidae^66^, and Terebellida^59,67^. On the other hand, fewer than seven travisiid species have been included in a molecular phylogeny^36,37,44,47^, and intra-familial relationships are not yet sufficiently discussed. *Travisia* is one of the most interesting subjects for evolutionary study as they inhabit a wide range of water depths and show a variety of morphological characters such as branchiae^34,35,41^. A phylogenetic analysis using more travisiid species would shed light on their evolution and diversification patterns in annelids in the future.

## Methods

### Sampling and DNA extraction

A specimen of *T. sanrikuensis* (GK627) was collected from 1659–1684 m depth in the northwestern Pacific (the Sanriku region, Japan) at 39°17′N, 142°48–49′E with a beam trawl during the cruise KS-17-12 of R/V *Shinsei-Maru*. The specimen was previously used as the non-type specimen of *T. sanrikuensis*^37^. Total DNA was extracted from body wall tissue of the fixed specimen in 70% ethanol using a DNeasy Blood and Tissue Kit (QIAGEN, Hilden, Germany) in the previous study. Extracted DNA was stored in a freezer at − 30 °C.

### Polymerase chain reaction and sequencing

Long PCR for the mitogenome of *T. sanrikuensis* was implemented following the method of Wu et al.^68^. A primer set for long PCR (Travi16SksF/Travi16SksR) (Table 4) was designed using the 16S rRNA sequence of *T. sanrikuensis* (GK627, GenBank accession number: LC566242). The PCR mixture for long PCR contained 14.0 μl of MilliQ water, 25.0 μl of 2 × Gflex PCR Buffer (TaKaRa, Shiga, Japan), 1.0 μl of 10 μM forward and reverse primers, 1.0 μl of Tks Gflex DNA Polymerase (TaKaRa), and 8.0 μl of template DNA solution. PCR amplification was performed as follows: 60 s at 94 °C for an initial denaturation, 36 cycles of 10 s at 98 °C, and 10 min at 68 °C. PCR product of > 15 kb in size was checked by electrophoresis in 1% agarose gel at 100 V for 40 min and then was used as a sample for next-generation sequencing. Bioengineering Lab. Co., Ltd., Japan, performed paired-end sequencing (2 × 151 bp) for the mitogenome ampliconusing an Illumina NextSeq 500 sequencer. Quality filtering for the sequences with a low-quality score (< 20) and short length (< 40) was performed using Sickle v1.33^69^.

**Table 4:** The primer sequences used in the present study.

Locus	Primer	Sequence (5′–3′)	Direction^a^	Usage^b^	Reference
16S rRNA	Trav16SksF	CTAATCCTCCTTAAGAGCCCATATTGACAGG	F	L	This study
	Trav16SksR	TTACTTTAGAGACAGATGGGCCTTCGTTTATCC	R	L	This study
*cox1*	LCO-annelid	CTCAACWAAYCAYAAAGAYATTGG	F	P/S	This study
	HCO2198	TAAACTTCAGGGTGACCAAAAAATCA	R	P/S	Folmer et al.^70^

^a^*F* forward, *R* reverse.

^b^*L* long PCR, *P* PCR, *S* sequencing.

A PCR primer LCO-annelid, which was modified from LCO1490^70^, was designed from the *cox1* gene sequences of annelids (see Table S1) and HCO2198^70^ were used to amplify *cox1* gene sequences of five *Travisia* spp. The PCR protocols for the *cox1* amplification of *Travisia* spp. (see Table S2 for GenBank accessions numbers) using KOD One PCR Master Mix (Toyobo, Tokyo, Japan), which is high efficiency for extension (5 s/kb for a target in 1–10 kb length), followed Kobayashi et al.^7^ except that 35 cycles, an annealing temperature of 50 °C, and an extension step of 20 s were used instead.

### Sequence analysis and gene annotation of the mitogenomes

Although the partial sequence of the 16S rRNA gene, which was not amplified by long PCR, was lacking in the NextSeq reads, a nearly complete mitogenome of *T. sanrikuensis* was assembled by NOVOPlasty v4.2.1^71^. First, NOVOPlasty assembly using the 16S rRNA gene sequence (LC566242) as a seed sequence was conducted with kmer and read length set to 23 bp and 111 bp, respectively. Then, another assembly was conducted with kmer and read length set to 39 bp and 151 bp, respectively. The seed for this second assembly was a partial sequence from the merged contig from the previous assembly. The nearly complete mitogenome of *T. sanrikuensis* was determined manually by concatenating the merged contig from the NOVOPlasty assembly result and the 16S rRNA gene sequence (LC566242). The PCGs were identified using the MITOS web server^72^. The positions of tRNAs were determined by the MITOS web server and ARWEN^73^, implemented in ARAGORN^74^. The secondary structures of tRNAs were predicted using ARAGORN. The annotated mitogenome sequence and raw reads are deposited in the DNA Data Bank of Japan (DDBJ) with DDBJ/EMBL/GenBank accession number LC677172 and DRA013124, respectivelly. Compositional skews were calculated as follows: AT-skew = (A − T)/(A + T), GC-skew = (G − C)/(G + C).

### Phylogenetic analysis based on mitogenomes

A preliminary phylogenetic analysis comprising the various lineages of annelid mitogenome sequences (149 OTUs) available from GenBank suggested that *T*. *sanrikuensis* is closely related to the clade of Arenicolida + Terebellida (Fig. S1 and Table S1). Based on this preliminary result and the results of a previous study^26^, 63 mitogenome sequences from a subset of Sedentaria (Arenicolida, Terebellida, echiurans, and clitellates), as well as two outgroups (Siboglinidae), were obtained from GenBank using the R package AnnotationBustR^75^ (Table 5). Outgroups were selected by referring to a review of annelid phylogeny^33^. *Erpobdella octoculata* (KC688270), *Hirudinaria manillensis* (KC688268), and *Hirudo nipponia* (KC667144) were indicated using double quotations and were excluded from discussion on phylogenetic relationships as Ye et al.^76^ suggested that species of these sequences were erroneously identified and should belong to *Whitmania*. DNA sequences of 13 PCGs were translated into amino acid (AA) sequences using the invertebrate mitochondrial genetic code with MEGA v7.0.26^77^. Alignment was performed using MAFFT v7 for AA sequences and two rRNA gene sequences (default parameters)^78^. PAL2NAL online service^79^ was used for codon alignments based on corresponding aligned AA sequences. Ambiguous positions were deleted with trimAl v.1.2^80^ with the -gappyout option.

**Table 5:** Mitochondrial genome sequences used in this study. Bold indicates the sequence obtained in the present study.

Group	Classification^a^	Family	Species^b^	GenBank accession No
Oligochaetes	Metagynophora			
		Moniligastridae	*Drawida japonica*	KM199288
	Crassiclitellata	Megascolecidae	*Amynthas aspergillus*	KJ830749
			*Amynthas carnosus*	KT429008
			*Amynthas corticis*	KM199290
			*Amynthas cucullatus*	KT429012
			*Amynthas gracilis*	KP688582
			*Amynthas hupeiensis*	KT429009
			*Amynthas jiriensis*	KT783537
			*Amynthas longisiphonus*	KM199289
			*Amynthas moniliatus*	KT429020
			*Amynthas morrisi*	KT429011
			*Amynthas pectiniferus*	KT429018
			*Amynthas robustus*	KT429019
			*Amynthas triastriatus*	KT429016
			*Amynthas* sp.	KT429010
			*Amynthas* sp.	KT429007
			*Amynthas* sp.	KT429014
			*Amynthas* sp.	KT429013
			*Duplodicodrilus schmardae*	KT429015
			*Metaphire californica*	KP688581
			*Metaphire guillelmi*	KT429017
			*Metaphire vulgaris*	KJ137279
			*Perionyx excavatus*	EF494507
			*Tonoscolex birmanicus*	KF425518
		Lumbricidae	*Aporrectodea rosea*	MK573632
			*Eisenia balatonica*	MK642872
			*Eisenia nana*	MK618511
			*Eisenia nordenskioldi nordenskioldi*	MK618509
			*Eisenia nordenskioldi nordenskioldi*	MK618510
			*Eisenia nordenskioldi nordenskioldi*	MK618513
			*Eisenia nordenskioldi nordenskioldi*	MK642867
			*Eisenia nordenskioldi nordenskioldi*	MK642868
			*Eisenia nordenskioldi pallida*	MK618512
			*Eisenia nordenskioldi pallida*	MK642869
			*Eisenia spelaea*	MK642870
			*Eisenia tracta*	MK642871
			*Lumbricus rubellus*	MN102127
			*Lumbricus terrestris*	U24570
		Rhinodrilidae^c^	*Pontoscolex corethrurus*	KT988053
Leeches	Arhynchobdellida			
	Hirudiniformes	Hirudinidae	*Hirudo medicinalis*	KU672396
			“*Hirudo nipponia*”	KC667144
			*Hirudo verbana*	KU672397
			“*Hirudinaria manillensis*”	KC688268
			*Whitmania acranulata*	KM655838
			*Whitmania laevis*	KC688269
			*Whitmania laevis*	KM655839
	Erpobdelliformes	Erpobdellidae	*Erpobdella japonica*	MF358688
			“*Erpobdella octoculata*”	KC688270
	Rhynchobdellida			
	Glossiphoniiformes	Glossiphoniidae	*Haementeria officinalis*	LT159848
			*Placobdella lamothei*	LT159849
			*Placobdella parasitica*	LT159850
	Oceanobdelliformes	Piscicolidae	*Zeylanicobdella arugamensis*	KY474378
		Ozobranchidae	*Ozobranchus jantseanus*	KY861060
Polychaetes		**Travisiidae**	***Travisia sanrikuensis* (GK627)**	**LC677172**
	Terebellida	Ampharetidae	*Auchenoplax crinita*	FJ976041
			*Decemunciger* sp.	KY742027
			*Eclysippe vanelli*	EU239687
		Alvinellidae	*Paralvinella sulfincola*	FJ976042
		Trichobranchidae	*Terebellides stroemii*	EU236701
		Terebellidae	*Pista cristata*	EU239688
		Pectinariidae	*Pectinaria gouldii*	FJ976040
	Arenicolida	Maldanidae	*Clymenella torquata*	AY741661
Echiurans	Capitellida	Thalassematidae^d^	*Urechis caupo*	AY619711
			*Urechis unicinctus*	EF656365
Outgroup	–	Siboglinidae	*Lamellibrachia luymesi*	KJ789163
			*Sclerolinum brattstromi*	KJ789167

^a^The classifications are after Jamieson^90^ for oligochaetes, Tessler et al.^53^ for leeches, and Struck^34^ for polychaetes.

^b^Double quotations indicate that species were possibly erroneously identified (see “Methods”).

^c^James^91^.

^d^Goto et al.^92^.

Phylogenetic trees were reconstructed based on the concatenated dataset using Bayesian inference and ML methods. Bayesian analysis was performed using Phylobayes 4.1^81^. Two parallel chains were made over 15,000 cycles using the CAT + GTR model. Convergence was automatically checked and terminated when maxdiff was < 1 and effective population size reached > 50 following the Phylobayes 4.1 manual. However, the run of AA dataset did not converge (maxdiff = 0.24 and effective population size < 50) after > 25,000 cycles and thus this tree was treated as supplementary data (Fig. S4). Phylogenetic trees using the ML method were reconstructed by IQ-TREE v1.6.12^82^ with 1000 ultrafast bootstrap replicates. Substitution models were selected with ModelFinder^83^ implemented in IQ-TREE. The resulting trees were edited using FigTree v1.4.3 (http://tree.bio.ed.ac.uk/software/figtree/).

### Intron analysis

In order to examine the phylogenetic relationships of the group II introns of *Travisia* and other annelids, phylogenetic analysis was conducted using a conserved region which consisted of domain V and subsequent sequences of the intron because the introns of *Travisia* spp. except for GK625 and GK1736 had no ORFs for putative proteins (i.e., reverse transcriptase or intron maturase). The *cox1* intron in the bivalve *Cucullaea labiata*^15^ was identified as group II in this study (see “Discussion”) and was included in the dataset. To find ORFs in the *Travisia* spp. intron, NCBI ORFfinder (https://www.ncbi.nlm.nih.gov/orffinder/) was used; then, all identified ORFs were used for searching protein domains in the Pfam-A collection of protein families by PfamScan (https://www.ebi.ac.uk/Tools/pfa/pfamscan/)^84^. A dataset for the phylogenetic analysis was built based on previous studies^16–18,85^, as shown in Table S2 and Dataset S1. Mfold web server online application RNA Folding Form V2.3 (http://www.unafold.org/mfold/applications/rna-folding-form-v2.php)^86^ was used to search the secondary structures of domain V and VI. The dataset was aligned using MAFFT with default options (resulted in 228 characters). The ML analysis was conducted by the same methods as mentioned above. The outgroup *Tetradesmus obliquus* (as *Scenedesmus obliquus* in Richter et al.^17^) was selected based on Richter et al. In total, 64 partial sequences of the group II intron were used for phylogenetic analysis because TreeShrink v1.3.9^87^ identified the *Clostridium difficile* sequence as a long branch, and it was excluded from the final dataset.

Sequences logos^88^ of the intron sequences, whose positions with gaps ≥ 20% were excluded, were generated using WebLogo^89^ to visualize the frequency of nucleotides of each position in the dataset. The sequence logos of introns of *Travisia*, except for GK1732 and GK1734 whose introns were not fully determined, were also created.

## Supplementary Information

Supplementary Figures.

Dataset S1.

Dataset S2.

Dataset S3.

Dataset S4.

Supplementary Tables.

## Data Availability

Assembled mitogenome, raw reads, and *cox1* sequences are available on NCBI repository (LC677172, LC670759–LC670765, DRA013124, PRJDB12599, SAMD00424219). Raw datasets generated during and/or analysed during the current study are available from the corresponding author on reasonable request.
