# Supplementary material for: Mitogenome of a stink worm (Annelida: Travisiidae) includes degenerate group II intron that is also found in five congeneric species
Source: Sci Rep. 2022 Mar 15;12:4449. doi: 10.1038/s41598-022-08103-5 (PMC8924214; doi:10.1038/s41598-022-08103-5)
Supplement: Supplementary file 1 — Supplementary Figures. [file 41598_2022_8103_MOESM1_ESM.pdf]

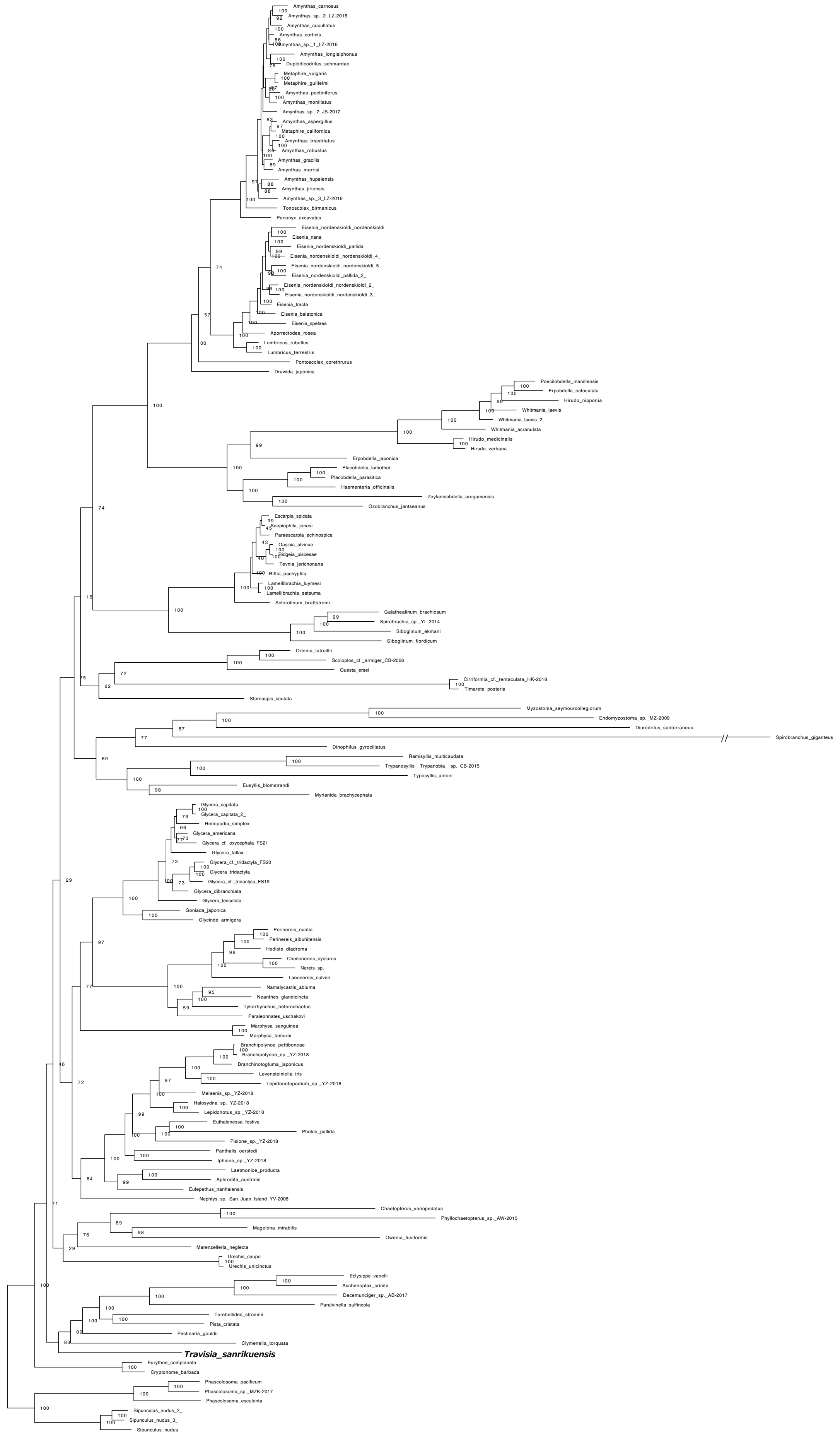

Figure S1. Preliminary analysis of maximum likelihood phylogeny of PCGs (2043 amino acids) of annelids (1000 bootstrap replicates). GenBank accession numbers are shown in Table S1. *Travisia sanrikuensis* is shown in bold.

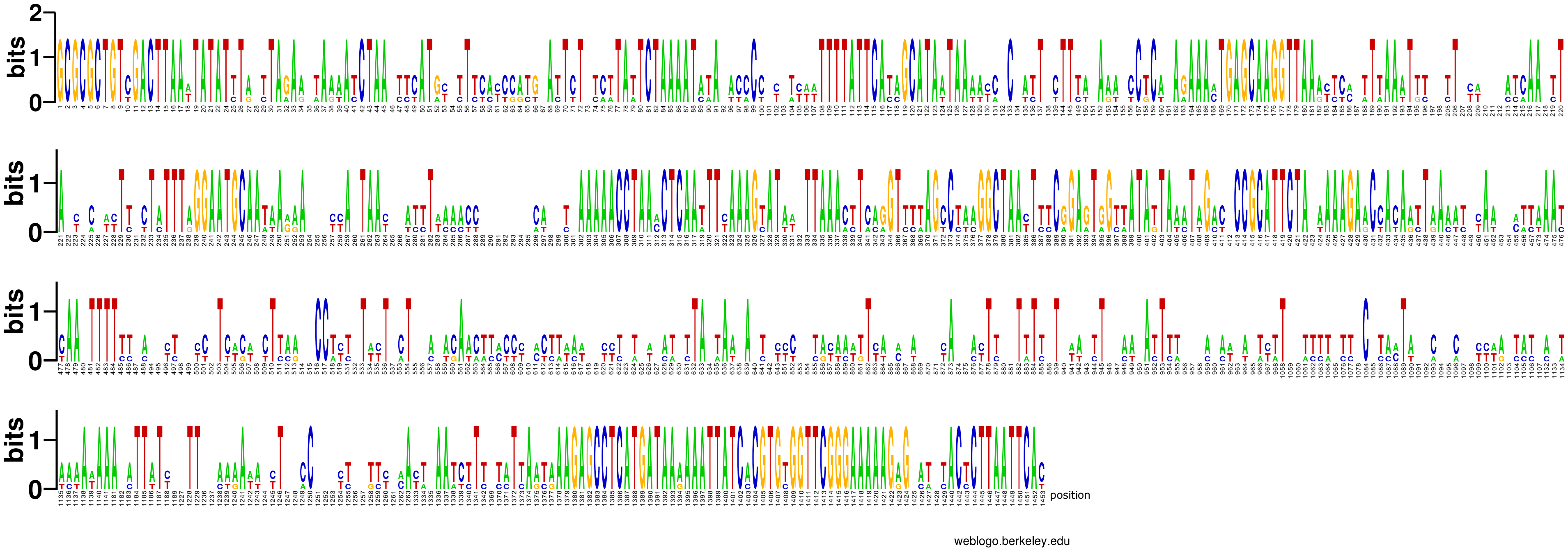

Figure S2. Sequence logos of the group II intron sequences of species of *Travisia*. Species in supplementary data 3 except for GK1732 and GK1736 were used as input data after positions including gaps ≥20% were excluded. The position numbers indicate the positions before gaps were trimmed, i.e., the numbers correspond to the positions of supplementary data 3.

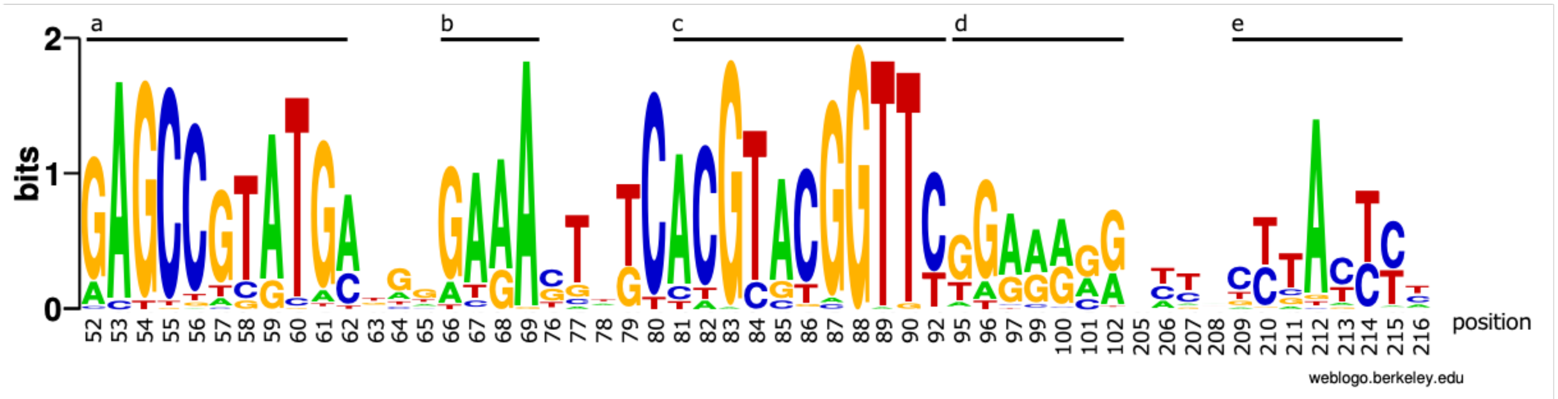

Figure S3. Sequence logos of the group II intron sequences. Supplementary data 1 was used as input data after positions including gaps ≥20% were excluded. The position numbers indicate the positions before gaps were trimmed , i.e., the numbers correspond to the positions of supplementary data 1. The lines indicate positions that roughly correspond to the stem (a & c) and ζ' (b) in the loop of domain V and the stem of domain VI (d & e).

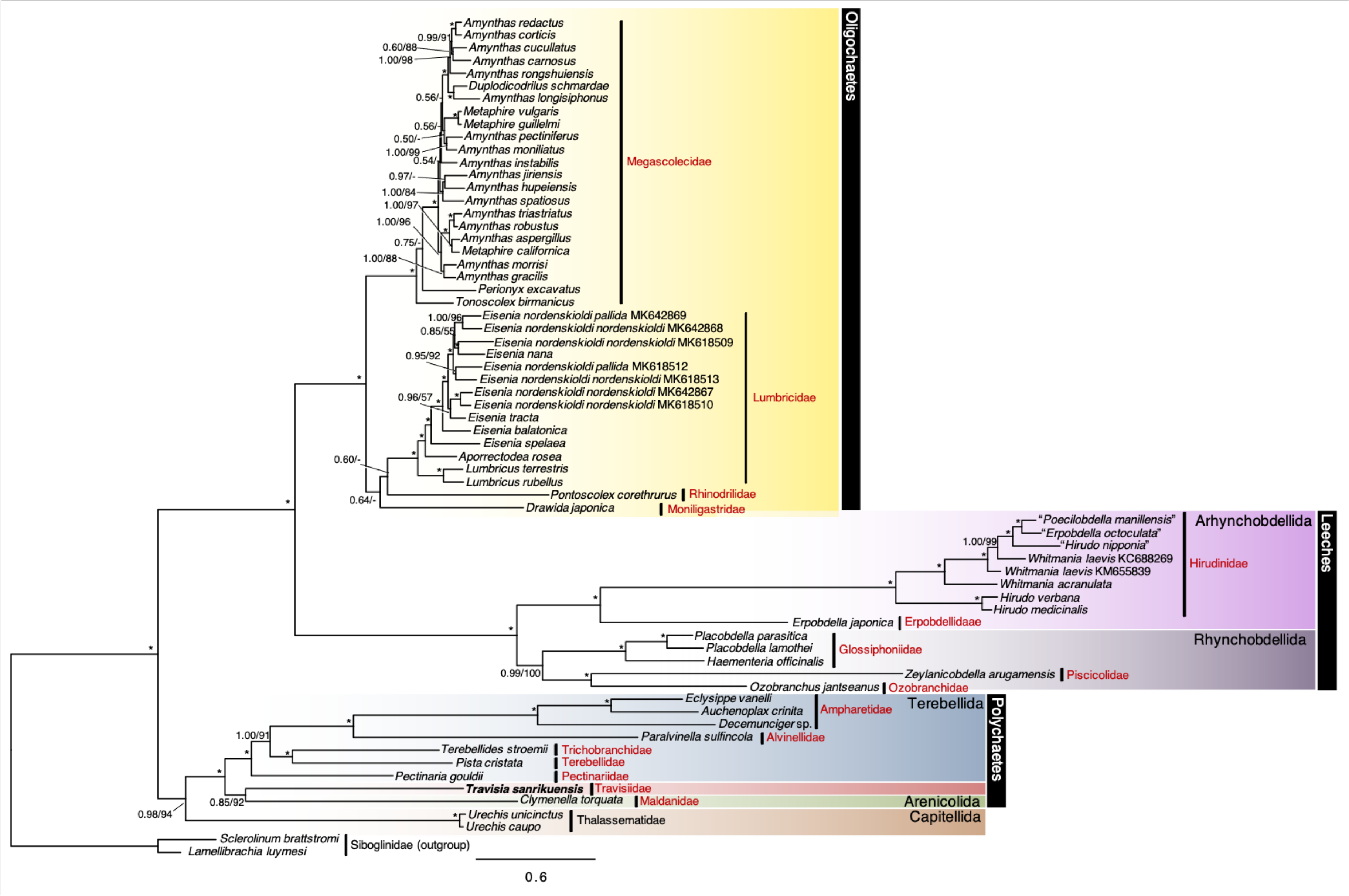

Figure S4. Bayesian phylogeny of the part of Sedentaria based on the concatenated dataset including amino acid sequences of 13 mitochondrial genome PCGs (4142 characters). Posterior probability (PP) followed by the percentage of the maximum likelihood bootstrap values (BS) above 50% is shown as numbers above branches. Asterisks indicate PP = 1 and BS = 100. *Travisia sanrikuensis*, whose nucleotide sequence was newly obtained, is shown in bold.
